# Symmetric Key Structural Residues in Symmetric Proteins with Beta-Trefoil Fold

**DOI:** 10.1371/journal.pone.0014138

**Published:** 2010-11-30

**Authors:** Jianhui Feng, Mingfeng Li, Yanzhao Huang, Yi Xiao

**Affiliations:** 1 Biophysics and Molecular Modeling Group, Department of Physics, Huazhong University of Science and Technology, Wuhan, China; 2 Department of Neurobiology and Kavli Institute for Neuroscience, Yale University School of Medicine, New Haven, Connecticut, United States of America; National Institute for Medical Research, Medical Research Council, London, United Kingdom

## Abstract

To understand how symmetric structures of many proteins are formed from asymmetric sequences, the proteins with two repeated beta-trefoil domains in Plant Cytotoxin B-chain family and all presently known beta-trefoil proteins are analyzed by structure-based multi-sequence alignments. The results show that all these proteins have similar key structural residues that are distributed symmetrically in their structures. These symmetric key structural residues are further analyzed in terms of inter-residues interaction numbers and B-factors. It is found that they can be distinguished from other residues and have significant propensities for structural framework. This indicates that these key structural residues may conduct the formation of symmetric structures although the sequences are asymmetric.

## Introduction

Symmetric proteins [1] are ideal objects to investigate protein evolution and folding. It is generally accepted that symmetric proteins have been arisen from gene duplications and fusions [2], [3]. However, these repetitive or symmetric signals were almost lost in their sequences during evolution but remain in their structures. Investigating how these proteins keep their symmetric structures by “asymmetric” sequences is a way to understand protein evolution and folding. On the other hand, understanding the building principle of symmetric proteins is also necessary for designing de novo proteins, because symmetric structures are relatively simple to be built from basic units. One solution to the problem above is that protein sequences may contain hidden symmetric signals that determine their symmetric structures [4]–[8]. Recently, we suggested that these hidden symmetric signals might be contributed by a small number (about 30%) of identical or key residues [9]–[15].

Multi-domain proteins provide ideal models to study the problem above since many of them consist of more than one domains evolved from the same ancestor and have similar structural symmetry but different sequence symmetry. For example, *Ricin Toxin B* (RTB, PDB id: 2aaib) is composed of two domains with the same beta-trefoil structure of three-fold symmetry [16]–[18]. It was speculated that RTB is the twice triplicate duplications of its ancestor, a galactose-binding peptide of about forty residues [18]. Rutenber *et al.* detected hidden three-fold sequence symmetry in both domains [18] but the degrees are very different. In its first domain the averaged sequence similarity index between the trefoil units equals 1.73 while in its second domain it is 2.63, i.e., one half larger than that of the first domain. This appears in contradiction with their almost identical structures. Since these two domains have evolved from the same ancestor, they are ideal model to understand sequence-structure relations of proteins. In fact, for RTB, Haze detected a three-fold repetitive QXW motif in both domains and regarded them as key structural residues [19]. Rutenber and Robertus also described a 12-residue hydrophobic core in both domains [20] and later Murzin *et al.* further showed that these residues are characteristic of the beta-trefoil fold [17]. It seems that these key residues may be the main factor to determine the symmetric structure. However, more evidences are needed to validate this conclusion. At least, we need to investigate other proteins in the same family.

According to *Structural Classification Of Proteins* (SCOP) databank [21], RTB belongs to *Plant Cytotoxin B-chain* (PCB) family and all proteins in this family contain two domains with beta-trefoil structure (see Materials and Methods). In this paper we shall analyze their sequence symmetries and identify their key structural residues by three different methods: structure-based multi-sequence alignments, residue interaction number and B-Factor analysis. We shall also extend our analysis to all presently known beta-trefoil proteins. Our results show that there exist similar key structural residues in all these proteins that may determine the symmetry of their structures.

## Materials and Methods

### Plant Cytotoxin B-chain Family

According to SCOP1.69, there are five species and sixteen protein chains in PCB family (Table 1). Among them, two species, *European mistletoe* and *Sambucus ebuLus*, have more than one protein chains. We select 1m2tb and 1hwmb as their representatives because both have crystal structures of the highest experimental resolutions (Table 1) [22]. The atomic coordinates of the crystal structures (PDB file) and experimental resolutions are retrieved from Protein Data Bank (Table 1).

**Table 1:** Characteristics of Plant Cytotoxin B-chain family.

Species	Protein Chain^a^	Resolution^b^(Å)	RMSD^c^(Å)
Castor bean	**2aaib**	2.50	1.50
Abrus precatorius	**1abrb**	2.14	1.24
MongoLian snake-gourd	**1ggpb**	2.70	1.77
European mistLetoe	**1m2tb**, 1pc8b, 1onkb, 1puub,		
1pumb,1oqlb, 1tfmb, 1ce7b, 2mllb	1.89	1.30	
Sambucus ebuLus	**1hwmb**,1hwob, 1hwnb, 1hwpb	2.80	1.50

a Bold entries indicate representative protein chains.

b Experiment resolution of crystal structure for representative protein chains.

c RMSD of structural superposition between domains for representative protein chains.

### Detection and Quantification of Protein Sequence Symmetry

In a previous paper [12], we developed a modified recurrence plot (MRP) algorithm to detect protein sequence symmetry, and defined two parameters *R* and *S* to quantify the degree of the detected sequence symmetry. Here, we only introduce them briefly.

The MRP of a protein sequence *x_1_ x_2_ x_3_… x_N_* is built as follows: the horizontal axis *i* denotes the location of the first residue of a segment in sequence and the vertical axis *d* denotes the length of the segment. For any segment *X_i_ = x_i_ x_i+1_ … x_i+d−1_*, if the number of its non-overlapping similar segments *X_j_ = x_j_ x_j+1_ … x_j+d−1_* (|*j−i|≥d*) is larger than the degree of symmetry you want to find, we plot a point at (*i*, *d*). The MRP is formed when this is done for all possible *i* and *d*. Two segments are similar if the percentage of their similar residues, obtained by using pair-wise global sequence alignment with PAM250 score matrix, is larger than a chosen number *r* and when p-value is lower than 0.05.

The parameter *R* is the Pearson's correlation coefficient between *i*MRP and *r*MRP, where *i*MRP denotes the ideal symmetric MRP corresponding to the real MRP (*r*MRP) of protein sequence. *R* reports the presence of non-overlapping repetitive patterns. Because the *R* value cannot definitely tell us the degrees of similarities of different patterns and so the degree of sequence symmetry, we introduce a parameter *S* to do this. *S* is the average value of the Pearson's correlation coefficients between all different patterns and describes the average similarity of different patterns. Therefore, the *S* value is a measure of the degree of sequence symmetry. For a sequence to be symmetric, both *R* and *S* should have large values. The details of this method can be found in ref. 12. It is noted that there existed other methods to find repeats of a protein sequence [4]–[8].

### Evaluation of Residue Interactions

The residue interaction number (RIN) of a residue is the number of the interaction pairs between this residue and other residues that are more than four residues apart along sequence and their potential energies are lower than −0.5kcal/mol [23], [24]. The potential energy is calculated with all-atom force field and implicit solvent model (GB/SA) [25], [26]. It is the sum of three energy terms: Van der Waals energy, electrostatic energy and solvent polarized energy. The third term denotes electrostatic interactions ΔG_pol_ between the solute and solvent and is calculated by
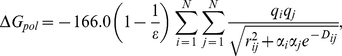
where 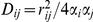 and *r_ij_* is the distance between atom *i* and atom *j*. *q_i_* and *q_j_* are the charges of atom *i* and atom *j*. *ε* is the dielectric constant of the solvent. α*_i_* is the effective Born radius of atom *i*, which is related to the effective Born free energy of solvation. The molecular mechanics software we used is Tinker with Charmm27 force field [27], [28]. Before formal calculations we optimize protein structure by conjugate-gradient method and the gradient tolerance is 0.1kcal/(Å mol).

## Results and Discussions

### Three-fold sequence symmetries of different degrees

**Figure 1:**
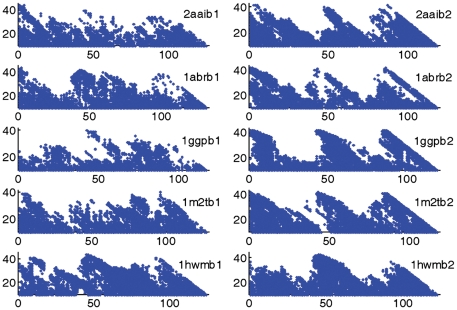
The MRPs of two domains in five representative protein chains. Column one is for the first domains and column two is for the second domains.

Fig. 1 gives the MRPs of the two domains of the five representative protein chains (*r* = 0.3 as in the previous paper [12]). It shows that all MRPs contain three repetitive patterns. The *R* values of all domains are larger than 0.5, and all the *S* values are larger than 0.4 only with one exception (Table 2). In our previous work, *R*≥0.5 and *S*≥0.4 are set as the cutoff values to measure whether a MRP shows symmetry or not [12]. Thus, almost all domains show hidden three-fold sequence symmetries. However, the MRPs of all the second domains reveal a pattern of three approximately right-angled triangles and the pattern is much more distinguishable than those of the first domains (Fig. 1). This means the symmetry degree of the second domains is higher than that of the first domains. In agreement with this, the *R* and *S* values of the second domains are all larger than those of the first domains with only one exception (Table 2) and the differences of the *S* values are significant, equaling 0.18, 0.10, 0.30, 0.22 and 0.18, respectively, and being about 35.3%, 22.7%, 54.6%, 34.4% and 34.6% of their respective means. This is in agreement with the result of RTB [18].

**Table 2:** Sequence symmetries for five representative protein chains.

Protein chains	Domain I		Domain II		ΔR^a^	ΔR/<R>^b^(%)	ΔS^a^	ΔS/<S>^b^(%)
	R	S	R	S				
2aaib	0.80	0.42	0.70	0.60	−0.10	−13.3	0.18	35.3
1abrb	0.73	0.39	0.75	0.49	0.02	2.7	0.10	22.7
1ggpb	0.69	0.40	0.73	0.70	0.04	5.6	0.30	54.6
1m2tb	0.64	0.53	0.72	0.75	0.08	11.8	0.22	34.4
1hwmb	0.66	0.43	0.75	0.61	0.09	12.8	0.18	34.6

a ΔR = RII−RI and ΔS = SII−SI;

b <R> = (RI+RII) and <S> = (SI+SII).

For the five representative proteins, the first domains are superposed to their second domains with the aid of OPAAS [29] and the root-mean-square distances (RMSD) are all less than 2Å (Table 1), i.e., the first and second domains have similar structures. Therefore, the symmetry degrees of the first and second domains are the same at structural level but different at sequence level. This is also in agreement with the result for RTB [18].

### Key structural residues of three-fold repetitions

#### Structure-based multi-sequence alignments

**Figure 2:**
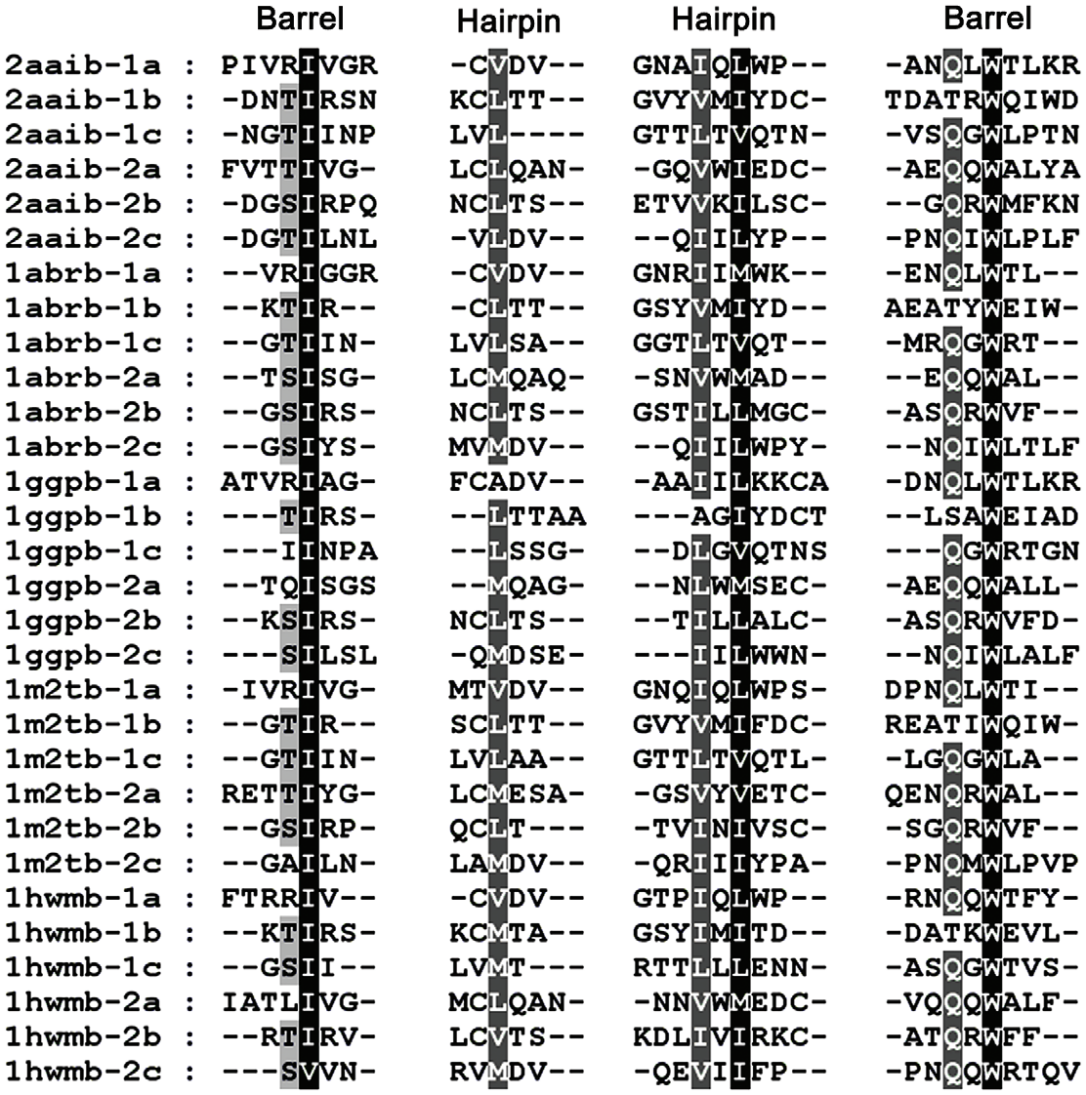
Structure based multiple sequence alignments of trefoil units in two domains of five representative protein chains. Conserved residues and most conserved residues are shaded gray and black respectively.

**Figure 3:**
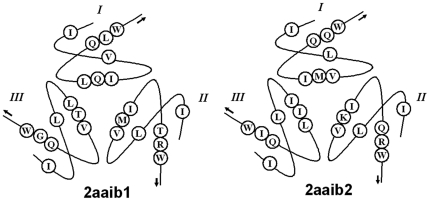
Schematic diagrams of four three-fold repetitive motifs (one-letter in circles) in two domains of RTB. The three trefoil units are shown in clockwise order. The arrows indicate the directions of beta strands.

In the first and second domains of all the five representative protein chains of PCB family, we identified four repetitive motifs through structure-based multi-sequence alignments of trefoil units (Fig. 2) [30], [31]. The repetitive motifs are (I)_3_, (L/M/V)_3_, ([I/L/V]X[I/L/M])_3_ and (QXW)_3_, where X denotes any residue. They are totally composed of twenty-four residues and show three-fold repetitions (Fig. 3). The four different residues (I, L, M, V) are all large hydrophobic residues [32], [33]. Generally, one residue is considered as buried if it has less than 25% solvent accessibility [34]. Using WHAT IF [35], we find that the four three-fold repetitive motifs are almost buried in the interior of their structures.

Consider RTB as an example to show the four three-fold repetitive (FTR) motifs in detail. The distribution of these motifs in the structure is illustrated in Fig. 3. It is shown that each beta strand has one motif and each trefoil unit has four motifs. Three-fold repetitions of the four motifs just correspond to the three-fold trefoil units in both domains. Moreover, these motifs are distributed symmetrically in the three-dimensional structures. The first motif is located at the top of the barrel structure, the fourth at the middle and the remaining two at the bottom. The FTR motifs seem to form the framework of the structures and act as key residues contributing to the formation of the symmetric structures, namely, the so-called key structural residues. Three previous works have reported some key structural residues in RTB [17], [19], [20]. Comparing them with the FTR motifs, we find they have a large overlap. Since other four representative protein chains show the same FTR motifs, they can be considered as the key structural residues of PCB family.

#### Inter-residue interactions

**Figure 4:**
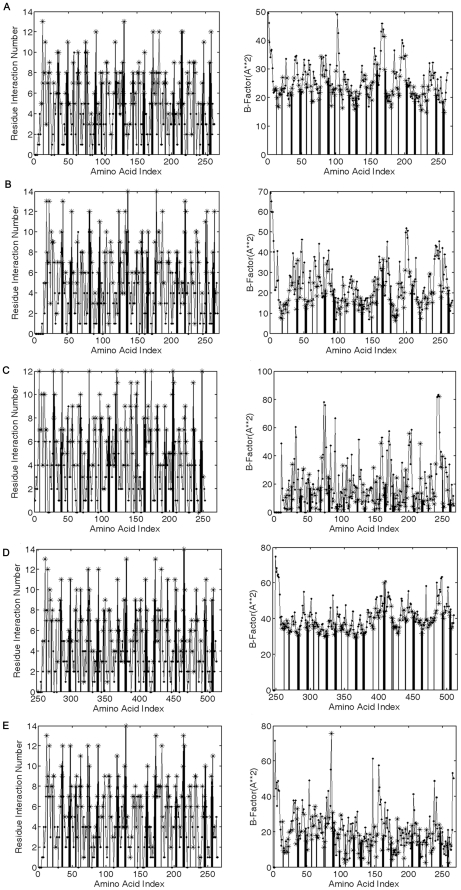
The residue interaction numbers (column one) and B-Factors (column two) versus amino acid index for 2aaib(A), 1abrb(B), 1ggpb(C), 1m2tb(D) and 1hwmb(E). The symbols represent different type of residues: four three-fold repetitive motifs (bar), buried residues (star) and remaining residues (dot).

We use another approach to confirm the FTR motifs acting as key structural residues in PCB family. We calculate their inter-residue interactions. The key structural residues should have more interactions with others. RTB is selected as an example too. The average residue interaction number (RIN) of all residues, buried residues, and all residues in FTR motifs is 4.98, 6.31 and 8.50 respectively (Table 3). The average RIN of the FTR motifs is the largest among them (Table 4). The FTR motifs are mainly composed of buried residues. Generally, a buried residue likely has a large RIN. However, the average RIN of the FTR motifs are larger than that of other buried residues. This indicates that they may play the role of key structural residues. Furthermore, as shown in the plot of the RIN versus amino acids, the residues in the FTR motifs almost always have the locally largest RINs although they may not be the globally largest (Fig. 4A). As for other four representative protein chains, the results are similar (Table 3 and Fig. 4). Hence, it is a common feature that the residues of the FTR motifs have larger RIN and they play the role of hubs in the inter-residue interaction network.

**Table 3:** The averaged residue interaction numbers and B-Factors.

Protein chains	Averaged RIN*			Averaged B-Factors*		
	A	B	R	A	B	R
2aaib	4.98	6.31	8.50	25.35	22.73	22.20
1abrb	5.08	6.33	8.92	23.12	18.00	17.26
1ggpb	4.82	6.18	8.33	19.32	14.61	11.68
1m2tb	4.81	5.95	8.79	40.55	37.03	36.51
1hwmb	5.10	6.03	8.92	20.88	16.52	16.37

*A-all residues, B-buried residues (eliminating buried residue in FTR motifs), R-FTR motifs.

**Table 4:** The averaged residue interaction numbers (RINs) for FTR motifs in five representative protein chains. The superscript numbers are their indices in sequences.

Protein chains	Trefoil unit	Motif I	RIN	Motif II	RIN	Motif III	RIN	Motif IV	RIN
**2aaib**	2aaib-1a	I^13^	7	V^21^	7	IQL^34–36^	9.33	QLW^47–49^	8
	2aaib-1b	I^57^	9	L^64^	10	VMI^75–77^	9.67	TRW^88–90^	8.67
	2aaib-1c	I^98^	7	L^105^	9	LTV^118–120^	8.33	QGW^129–131^	9.33
	2aaib-2a	I^144^	8	L^152^	8	VWI^159–161^	8	QQW^171–173^	8.33
	2aaib-2b	I^181^	8	L^191^	8	VKI^202–204^	7	QRW^214–216^	11
	2aaib-2c	I^224^	7	V^233^	9	IIL^245–247^	7.67	QIW^256–258^	8.33
**1abrb**	1abrb-1a	**I** ^18^	8	**V** ^26^	9	**IIM** ^39–41^	10	**QLW** ^52–54^	8
	1abrb-1b	**I** ^62^	9	**L** ^69^	8	**VMI** ^80–82^	10	**TYW** ^93–95^	8.33
	1abrb-1c	**I** ^103^	7	**L** ^110^	8	**LTV** ^123–125^	8.67	**QGW** ^134–136^	10
	1abrb-2a	**I** ^149^	8	**M** ^157^	10	**VWM** ^164–166^	7.67	**QQW** ^176–178^	9.33
	1abrb-2b	**I** ^186^	8	**L** ^196^	8	**ILL** ^207–209^	7.67	**QRW** ^219–221^	11.67
	1abrb-2c	**I** ^229^	7	**M** ^238^	9	**IIL** ^250–252^	9.67	**QIW** ^261–263^	8.67
**1ggpb**	1ggpb-1a	**I** ^18^	7	**A** ^26^	6	**IIL** ^39–41^	10	**QLW** ^52–54^	8
	1ggpb-1b	**I** ^62^	8	**L** ^69^	9	**AGI** ^81–83^	8	**SAW** ^93–95^	8
	1ggpb-1c	**I** ^104^	6	**L** ^112^	8	**LGV** ^123–125^	7	**QGW** ^134–136^	9.33
	1ggpb-2a	**I** ^149^	7	**M** ^157^	11	**LWM** ^164–166^	10	**QQW** ^176–178^	9
	1ggpb-2b	**I** ^186^	7	**L** ^196^	9	**ILL** ^207–209^	6.33	**QRW** ^219–221^	11
	1ggpb-2c	**I** ^229^	6	**M** ^238^	9	**IIL** ^250–252^	8.33	**QIW** ^261–263^	7.33
**1m2tb**	1m2tb-1a	**I** ^262^	7	**V** ^269^	7	**IQL** ^282–284^	9	**QLW** ^295–297^	7.67
	1m2tb-1b	**I** ^305^	8	**L** ^312^	10	**VMI** ^323–325^	10	**TIW** ^336–338^	8.67
	1m2tb-1c	**I** ^346^	8	**L** ^355^	8	**LTV** ^366–368^	7.67	**QGW** ^377–379^	9.33
	1m2tb-2a	**I** ^392^	9	**M** ^400^	9	**VYV** ^407–409^	8.33	**QGW** ^419–421^	9.67
	1m2tb-2b	**I** ^429^	8	**L** ^439^	11	**INI** ^450–452^	9	**QRW** ^462–464^	10.67
	1m2tb-2c	**I** ^472^	6	**M** ^481^	10	**III** ^493–495^	9	**QMW** ^504–506^	8
**1hwmb**	1hwm-1a	**I** ^15^	8	**V** ^23^	7	**IQL** ^36–38^	10.33	**QQW** ^47–49^	8.33
	1hwm-1b	**I** ^57^	8	**M** ^64^	11	**IMI** ^75–77^	10	**TKW** ^88–90^	8.33
	1hwm-1c	**I** ^98^	7	**M** ^107^	9	**LLL** ^118–120^	9	**QGW** ^129–131^	10.67
	1hwm-2a	**I** ^144^	6	**L** ^152^	7	**VWM** ^161–163^	8.33	**QQW** ^173–175^	9.67
	1hwm-2b	**I** ^183^	8	**V** ^193^	9	**IVI** ^204–206^	7.67	**QRW** ^215–217^	11.67
	1hwm-2c	**I** ^226^	6	**M** ^234^	9	**VII** ^246–248^	7.67	**QQW** ^257–259^	9.33

**Figure 5:**
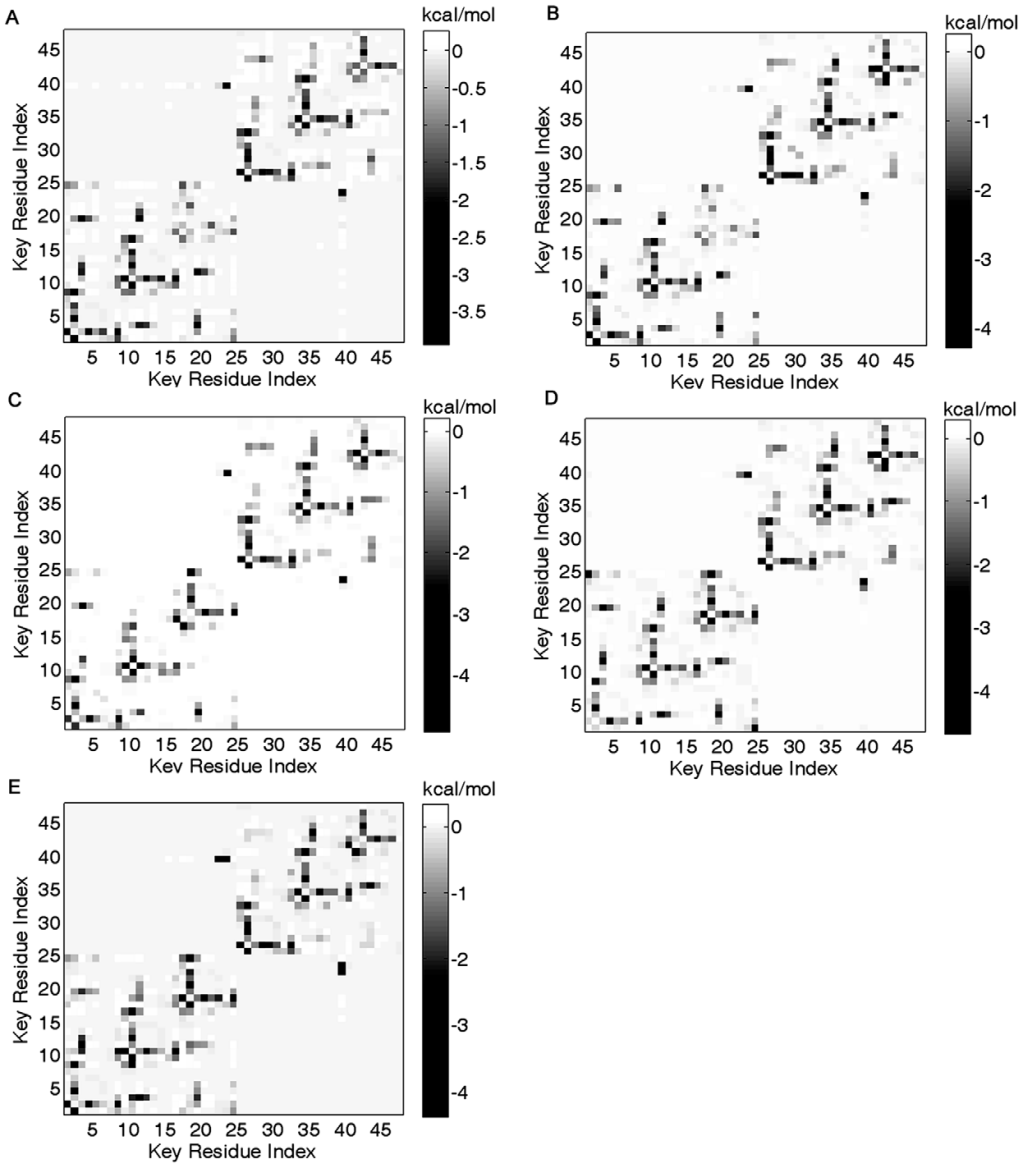
The potential energies of residue interactions between key structural residues for 2aaib(A), 1abrb(B), 1ggpb(C), 1m2tb(D) and 1hwmb(E). The key structural residues are arrayed along two axes according to their orders in the sequence. The magnitude of the interactions is indicated by the colorbar.

Fig. 5 gives the interaction energies between the key structural residues of each representative protein chain (Fig. 5). In each plot there are six “L”-like patterns along diagonal (each domain has three patterns), which denote the strong residue interactions. There are few interactions between different trefoil units. We compared these patterns with the positions of the key structural residues and found the six “L”-like patterns are just corresponding to the six repetitions of the four motifs or the six trefoil units. Furthermore, the “L”-like patterns indicate similar inter-residue interaction patterns in every trefoil unit. Therefore, every trefoil units not only have similar key structural residues but also similar strong residue interactions. This suggests that the repetitive key structural residues may determine the three-fold trefoil units. Finally, the “L”-like patterns show that the second motifs, (L/M/V)_3_, have stronger interactions with other motifs. This may be that the second motifs are closer to other three motifs (Fig. 3).

#### B-factors

From an experimental point of view, since the key structural residues act as the skeleton of structures, they should be much more constrained than other residues. The B-factors retrieved from PDB file are generally characteristic of the degree of atomic constraint. We average the B-factors of all heavy atoms in one residue and designate the mean as the B-factor of this residue. For RTB, the average B-factor of all residues, buried residues, and all residues in the FTR motifs is 25.35, 22.73 and 22.20 respectively (Table 3). Clearly, the FTR motifs have the smallest average B-factor. Furthermore, as shown in the plot of the B-factors versus amino acids, the residues in the FTR motifs always have the locally smallest B-factors (Fig. 4A). As for other four representative protein chains, we gain the same results as RTB (Table 3 and Fig. 4). Therefore, the FTR motifs seem to be most strongly constrained. In summary, both the inter-residue interactions and B-factors also suggest that the FTR motifs may be key structural residues in PCB family.

### Extension to all beta-trefoil folds

Are the three-fold repetitive key structural residues special for beta-trefoil proteins in PCB family or common for all proteins sharing beta-trefoil fold? In our recently published paper [12], thirty protein chains/domains were selected as the representatives of the presently known proteins with beta-trefoil fold. Because the two domains of 1vcla are homologous and also because only the atomic coordinates of alpha carbon atoms can be retrieved from PDB database for 2ila-, twenty-eight protein chains/domains are set as the representatives (Table S1 in Supporting file S1). Two algorithms, CE and TM-align integrated in STRAP [36]–[38], are used to do their structure-based multiple sequence alignments. Interestingly, both alignment methods detected similar twelve conserved motifs (Figure S1 and Figure S2 in Supporting file S1). We compare them with the FTR motifs and find they are similar. The twelve conserved motifs also show three-fold repetitions. In addition, we notice the twelve conserved residues as well as the FTR motifs are mainly composed of large hydrophobic residues (I, L, V, F, W), which is in agreement with the previous prediction by Murzin *et al.* that the large hydrophobic residues stabilize the beta-trefoil fold [17]. Recently, Chaudhuri *et al.* [39] pointed out that at least 80% propellers across families are similar at a level indicative of homology. To support their conclusion, one evidence is that all propellers share similar key sequence motifs across families. We [23], [24] also studied the key residues in the protein domain G from transducin (PDB id: 1tbg ), which is a propellerlike protein composed of seven similar blades or called WD-repeats and has a high structural symmetry. From a structure-based sequence alignment, it can be observed that there are five residues that are almost totally invariant in each repeat of the protein. These structurally conserved residues connect the outer strand of each blade to the inner three strands of the next blade, and are certainly considered as key residues critical for the structural stability of the G protein. We calculated the contact energies by all-atom force field and found that the residues with lowest contact energies (or strong inter-residue interactions) are in good agreement with the structurally conserved residues identified previously. Here, the proteins with beta-trefoil fold show the similar situation. All evidences suggest that the three-fold repetition of key structural residues should dominate the three-fold symmetric structures. Thus, the contradiction of different degrees of structure and sequence symmetries of the two domains of PCB family proteins can be interpreted in terms of similar key structural residues.

In conclusion, we analyzed the proteins with two repeated beta-trefoil domains in Plant Cytotoxin B-chain family and all presently known beta-trefoil proteins by three different methods and show that some key structural residues may play important roles in the formation of the three-fold symmetric structure of beta-trefoil fold. These key structural residues are (i) buried residues, (ii) symmetrically located in the structure, and (iii) have large residue interaction numbers and small B-Factors. This result may be helpful to design de novo proteins.

## Supporting Information

Supporting File S1 Supplementary data (Table S1; Figures S1, S2)
(3.50 MB DOC)
