# Supplementary material for: Symmetric Key Structural Residues in Symmetric Proteins with Beta-Trefoil Fold
Source: PLoS One. 2010 Nov 30;5(11):e14138. doi: 10.1371/journal.pone.0014138 (PMC2994741; doi:10.1371/journal.pone.0014138)
Supplement: Supporting File S1 — Supplementary data (Table S1; Figures S1, S2) (3.50 MB DOC) [file pone.0014138.s001.doc]

# Symmetric Key Structural Residues in Symmetric Proteins with Beta-trefoil Fold

Jianhui Feng[[1]](#footnote-2)+, Mingfeng Li[[2]](#footnote-3)+ , Yanzhao Huang and Yi Xiao[[3]](#footnote-4)*

*Biophysics and Molecular Modeling Group, Department of Physics, Huazhong University of Science and Technology, Wuhan 430074, Hubei, China*

**Supplementary data (Tables S1; Figures S1- S2)**

**Proteins with beta-trefoil fold**

According to SCOP1.69, proteins (protein chains or domains) in forty-five species (included in eight superfamilies and twelve families) are categorized to the beta-trefoil fold. One representative protein is selected from each species. These selected proteins cover monomeric and oligomeric proteins. For an oligomeric protein, only one chain is selected provided that more than one chain shares beta-trefoil fold. Aside from them, nine protein chains are composed of multiple domains sharing the same beta-trefoil fold, e.g. 1jlxa. Taken together, we get fifty-six proteins with beta-trefoil fold (Table S1).

We use ClustalW to filter these proteins with sequence identity larger than 30%, and thirty-three proteins are remaining (Table S1). Besides, four protein domains (PDB id’s: 1a8d-2, 3btaa1, 1epwa4 and 1ttua1) having relatively weak structural symmetry will be eliminated, evaluation aided by OPAAS method. Also, one protein (PDB id: 2ila-) is not considered because there are only the atomic coordinates of its alpha carbon atoms in PDB database. So, twenty-eight proteins will finally be selected as the representations of the known proteins sharing beta-trefoil fold.

Table S1. The thirty-three non-homologous proteins representing of the fifty-six proteins selected from each species of the beta-trefoil fold

| Protein | Location | Representation | Protein | Location | Representation |
| --- | --- | --- | --- | --- | --- |
| 1bfg- | - | 1bfg- | 1xyfa2 | A:313-436 | 1knm- |
| 1bara | A |  | 1knm- | - |  |
| 1jqza | A |  | 1qxma1 | A:4-148 | 1qxma1 |
| 1fmms | - |  | 1qxma2 | A:149-286 | 1qxma2 |
| 1ijta- | - |  | 1sr4a | A | 1sr4a |
| 1qqka | A |  | 1sr4c | C | 1sr4c |
| 1ihk- | - |  | 1vcla1 | A:1-150 | 1vcla1 |
| 1nuna | A |  | 1vcla2 | A:151-283 |  |
| 1q1u- | - |  | 1dqg- | - | 1dqg- |
| 1pwa- | - | 1pwa- | 1jlxa1 | A:1-153 | 1jlxa1 |
| 1l2h- | - | 1l2h- | 1jlxa2 | A:154-299 | 1jlxa2 |
| 8i1b- | - |  | 1wba- | - | 1tie- |
| 1ilr1 | 1 | 1ilr1 | 1tie- | - |  |
| 1md6- | - |  | 1eyl- | - |  |
| 2ila- | - | 2ila- | 1avwb | B |  |
| 1j0s- | - | 1j0s- | 1avac | C | 1avac |
| 1n4ka1 | A:236-435 | 1n4ka1 | 1r8n- | - | 1r8n- |
| 1t9f- | - | 1t9f- | 1r8o- | - | 1r8o- |
| 2aaib1 | B:1-135 | 2aaib1 | 1a8d-2 | 248-452 | 1a8d-2 |
| 1abrb1 | B:1-140 |  | 3btaa1 | A:1079-1295 | 3btaa1 |
| 1ggpb1 | B:11-140 |  | 1epwa4 | A:1079-1290 | 1epwa4 |
| 1m2tb1 | B:249-384 |  | 1dfca1 | A:1008-1140 | 1dfca1 |
| 1hwmb1 | B:3-135 |  | 1dfca2 | A:1141-1259 | 1dfca2 |
| 2aaib2 | B:136-262 | 2aaib2 | 1dfca3 | A:1260-1382 | 1dfca3 |
| 1abrb2 | B:141-267 |  | 1dfca4 | A:1383-1493 | 1dfca4 |
| 1ggpb2 | B:141-267 |  | 1hcd- | - | 1hcd- |
| 1m2tb2 | B:385-510 |  | 1ttua1 | A:381-541 | 1ttua1 |
| 1hwmb2 | B:136-266 |  | 1wd3a2 | A:338-499 | 1wd3a2 |


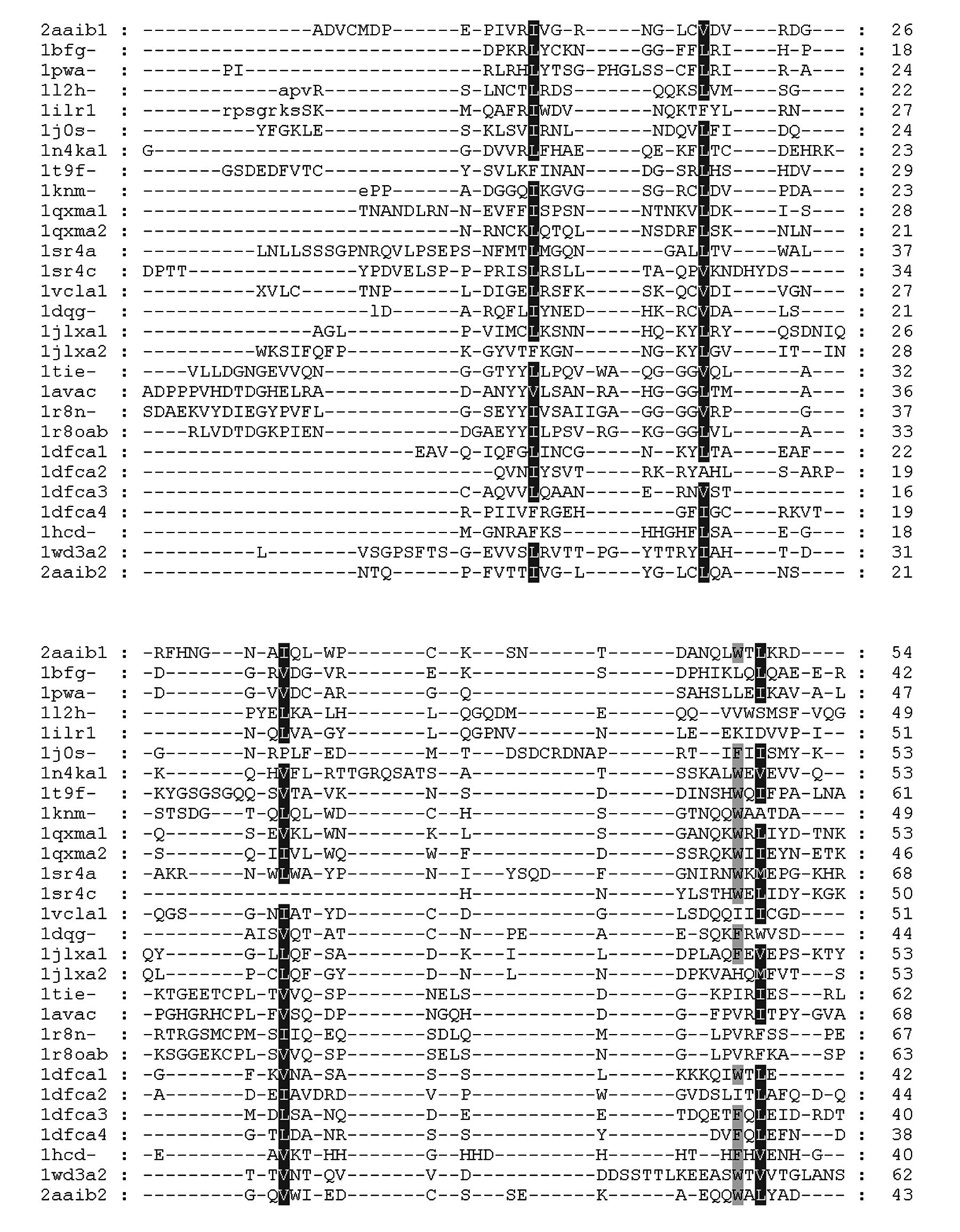

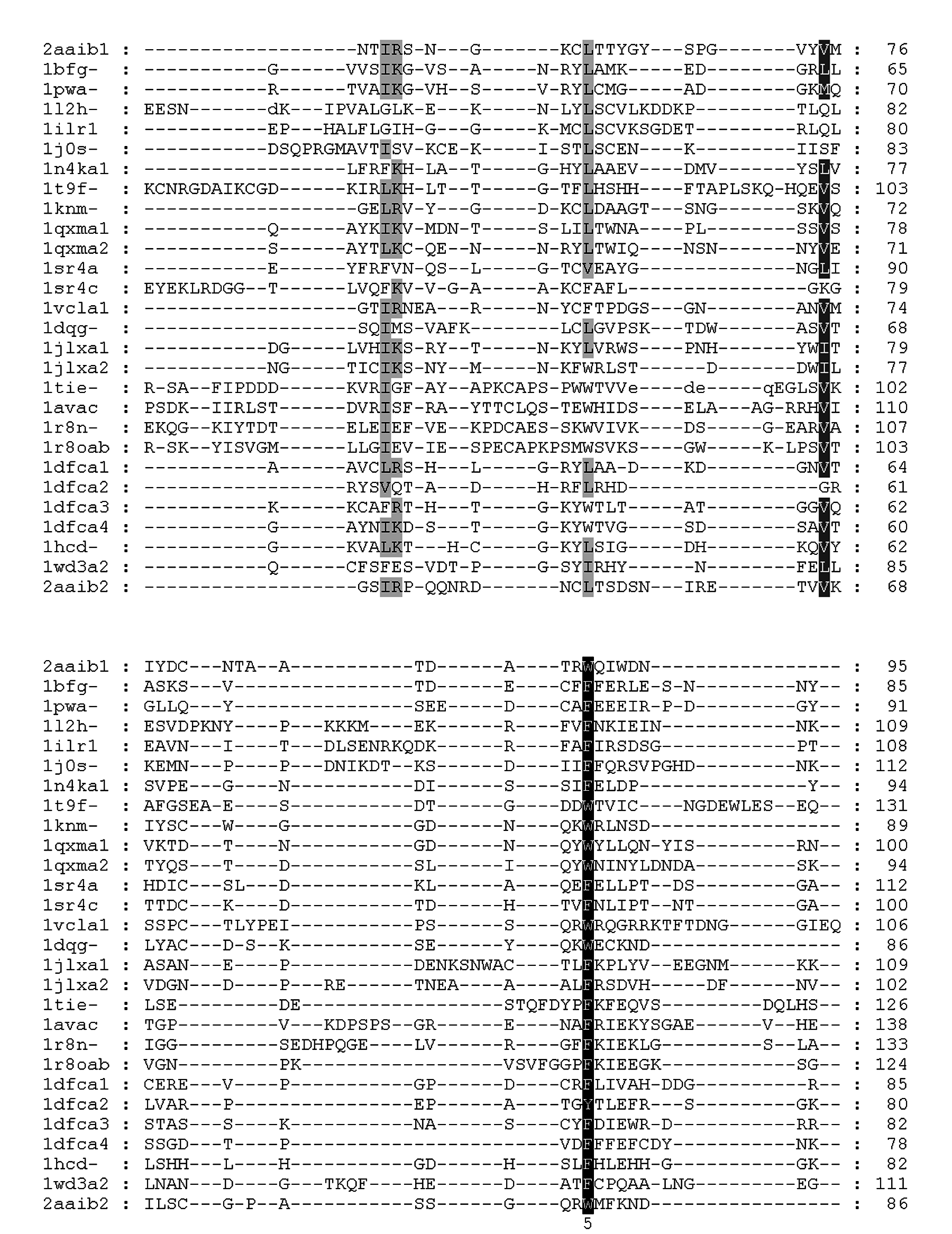

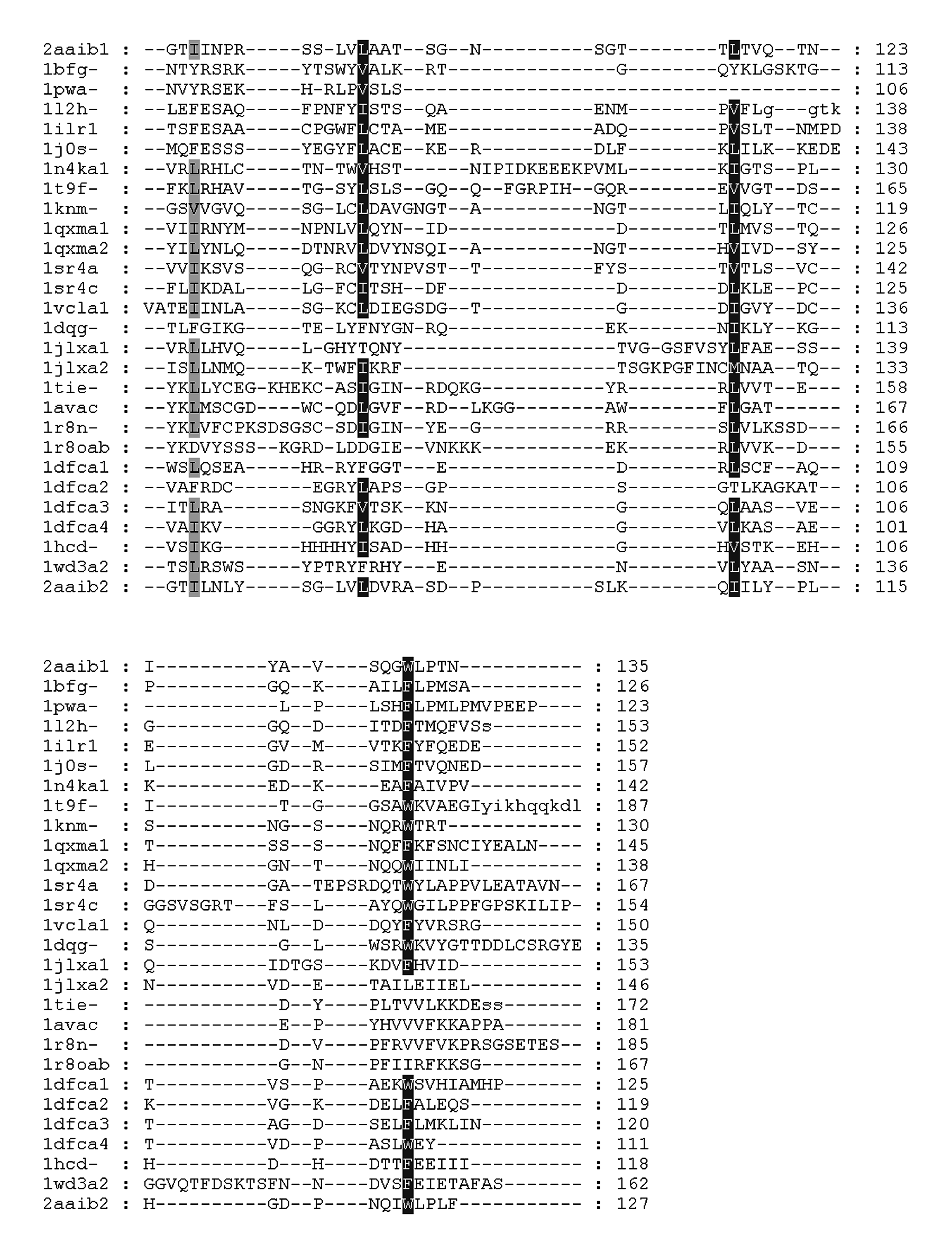


Fig.S1. Structure aided sequence alignments of the twenty-eight representative proteins by STRAP through CE algorithm. Conserved residues and most conserved residues are shaded gray and black respectively.


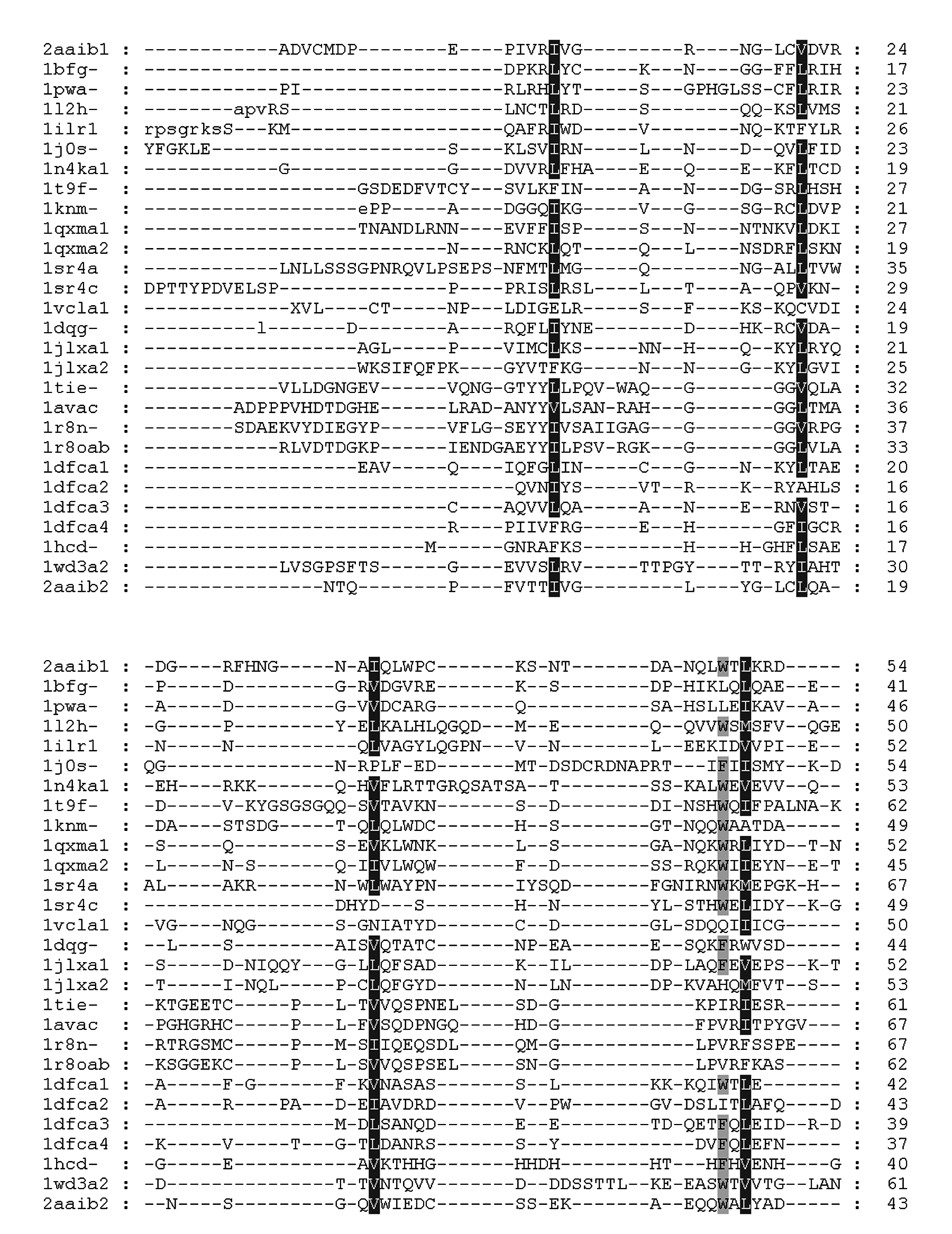

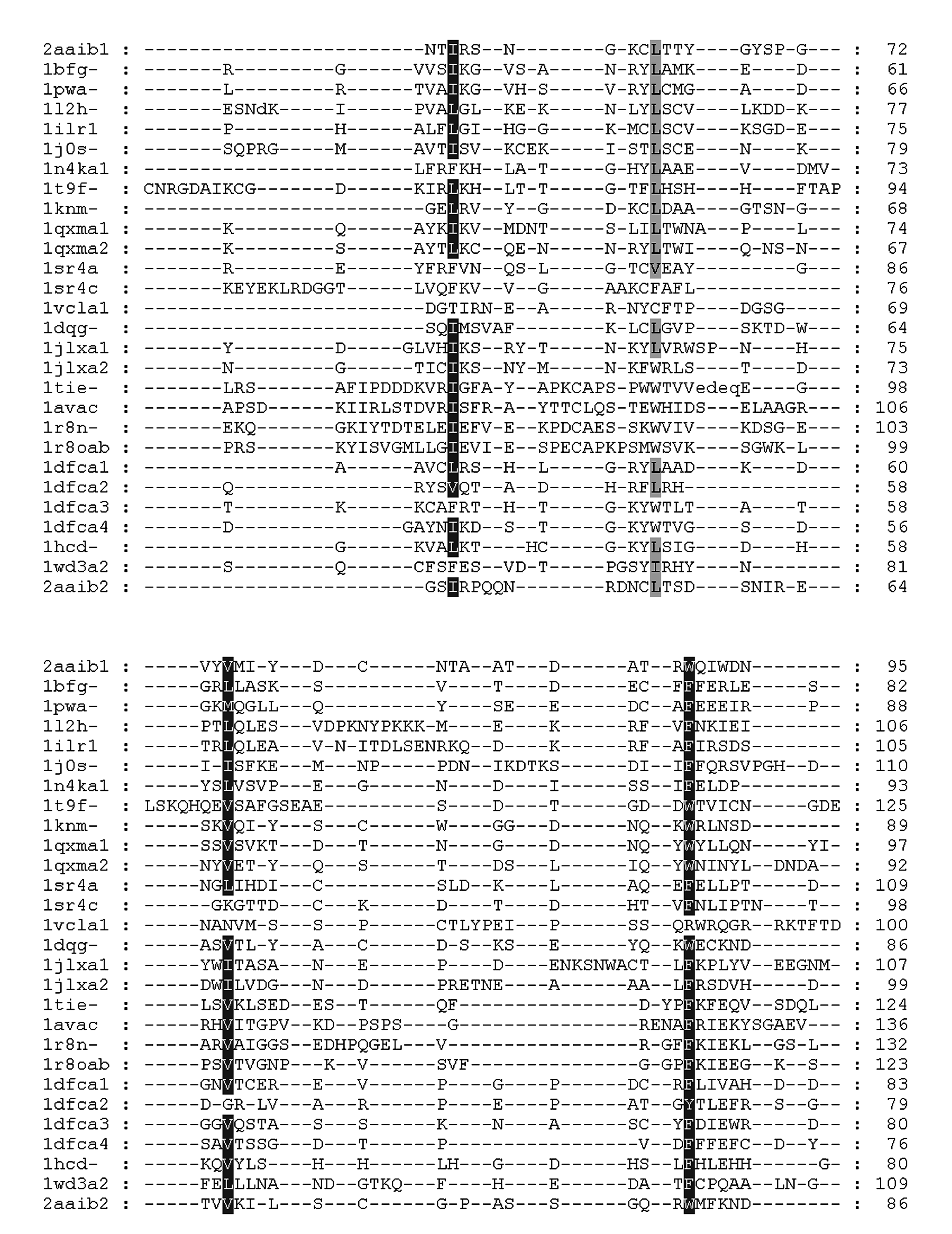

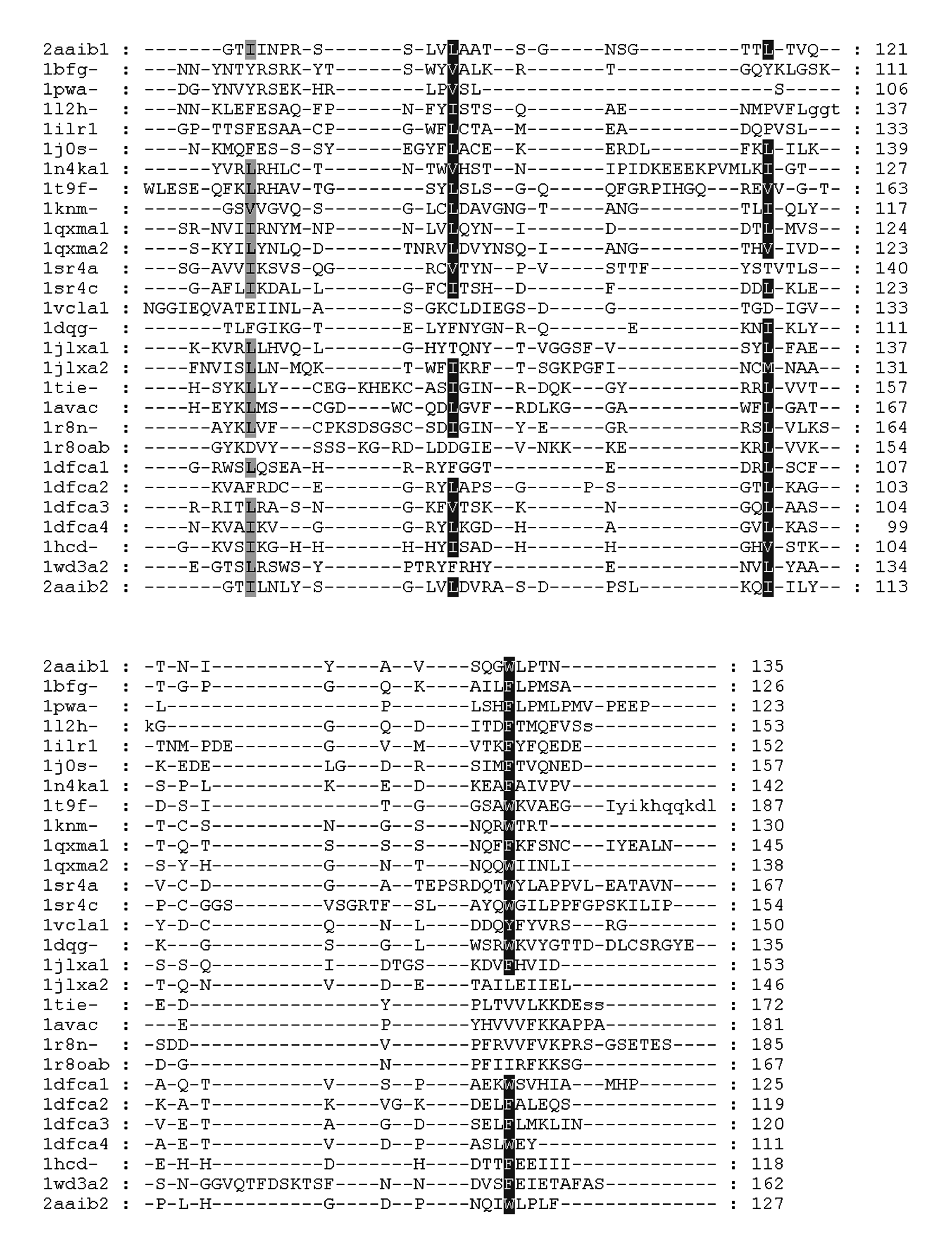


Fig.S2. Structure aided sequence alignments of the twenty-eight representative proteins by STRAP through TM-align algorithm. Conserved residues and most conserved residues are shaded gray and black respectively.

1. ^+^ Co-first authors. [↑](#footnote-ref-2)
2. ^+^ [↑](#footnote-ref-3)
3. ^*^ Corresponding author. E-mail: yxiao@mail.hust.edu.cn. [↑](#footnote-ref-4)
